# A202 IMPACT OF THE COVID-19 PANDEMIC ON THE EPIDEMIOLOGY OF ALCOHOL-RELATED HEPATITIS

**DOI:** 10.1093/jcag/gwab049.201

**Published:** 2022-02-21

**Authors:** A Frolkis, M Borman, M D Sadler, S E Congly, H H Nguyen, S Lee, L Stinton, M Swain, C S Coffin, A Aspinall, K W Burak, A M Shaheen

**Affiliations:** 1 University of Calgary, Calgary, AB, Canada; 2 Division of Gastroenterology, University of Calgary, Calgary, AB, Canada; 4 Liver Unit, Division of Gastroenterology, Department of Medicine, University of Calgary Cumming School of Medicine, Calgary, AB, Canada; 5 Gastroenterology and Hepatology, University of Calgary, Calgary, AB, Canada; 8 Medicine, University of Calgary, Calgary, AB, Canada; 10 Liver Unit, Univ Calgary, Calgary, AB, Canada

## Abstract

**Background:**

Alcohol-related hepatitis (AH) is the most severe form of alcohol-related liver disease, with rising incidence. Stay-at-home orders for the COVID-19 pandemic were associated with increased alcohol consumption. Online sales reported a 262% increase from March 2019 to 2020.

**Aims:**

The purpose of this study was to track the epidemiology of hospitalizations for AH by sex before and after the COVID-19 pandemic. We hypothesized that AH would be more severe in females and younger individuals during the pandemic.

**Methods:**

Using the Discharge Abstract Database, we identified all hospitalizations in Alberta with international classification of disease-10 codes for AH between March 2018 and September 2020. We merged this dataset with provincial laboratory data to identify all inpatient lab values. We calculated Model for End-Stage Liver Disease (MELD) and Maddrey scores and validated a laboratory-based algorithm for AH. Severe AH was defined as Maddrey score > 32. Onset of the pandemic was defined as March 2020. Stratified by pandemic onset, descriptive statistics were done with Chi-squared and Kruskal Wallis tests. Inpatient mortality was assessed as a primary outcome. Binomial regression was used to assess changes in frequency of admission for AH with the denominator as all cirrhosis-related admissions over the same time-period.

**Results:**

We identified 991 hospitalizations for AH prior to the pandemic (n=381, 38.5% female) and 417 during the pandemic (n=144, 34.5% female). Hospitalizations for AH significantly increased during the pandemic (p = 0.04) (Figure 1). Median Maddrey score for females (30.5) before the pandemic was significantly higher than for males (22.9), p < 0.01. During the pandemic, median Maddrey for females (28.7) was higher than males 21.4, p = 0.07. Median age at admission was significantly lower for both males and females during the pandemic (age 44 and 41, respectively) as compared to prior (age 47 and 45, respectively) p < 0.05. There was no significant difference in MELD between sexes before (13.5 for females, 14.0 for males, p = 0.15) and during the pandemic (13.3 for females, 13.0 for males, p = 0.75). Additionally, there was no significant difference in mortality between sexes before (10.4% in females, 11.5% in males, p = 0.22) and after the pandemic (9.2% in females, 9.9% in males, p = 0.67).

**Conclusions:**

Hospitalizations for AH rose during the pandemic and occurred at younger ages. There was no significant difference in disease severity or mortality before and during the pandemic. Overall, females have more severe AH than males. Public health efforts should continue to be made to educate about the harms of alcohol excess and offer community support. Future studies will expand the trend through multiple pandemic waves.

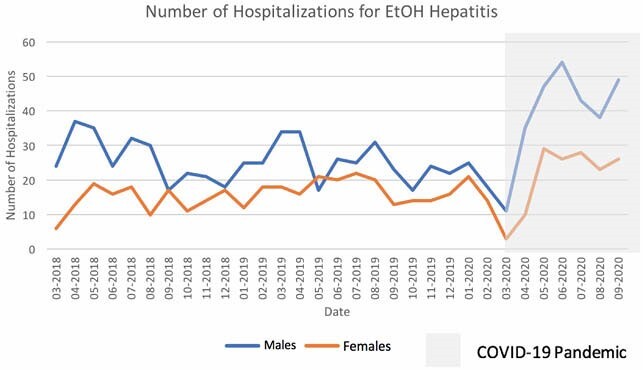

**Funding Agencies:**

None

